# Global spatio-temporally harmonised datasets for producing high-resolution gridded population distribution datasets

**DOI:** 10.1080/20964471.2019.1625151

**Published:** 2019-06-18

**Authors:** Christopher T. Lloyd, Heather Chamberlain, David Kerr, Greg Yetman, Linda Pistolesi, Forrest R. Stevens, Andrea E. Gaughan, Jeremiah J. Nieves, Graeme Hornby, Kytt MacManus, Parmanand Sinha, Maksym Bondarenko, Alessandro Sorichetta, Andrew J. Tatem

**Affiliations:** a WorldPop, School of Geography and Environmental Science, University of Southampton, Southampton, UK; b Flowminder Foundation, Stockholm, Sweden; c Center for International Earth Science Information Network (CIESIN), Columbia University, Palisades, NY, USA; d Department of Geography and Geosciences, University of Louisville, Louisville, KY, USA; e GeoData, University of Southampton, Southampton, UK

**Keywords:** Human population, sub-national, global, spatial dataset, multi-temporal

## Abstract

Multi-temporal, globally consistent, high-resolution human population datasets provide consistent and comparable population distributions in support of mapping sub-national heterogeneities in health, wealth, and resource access, and monitoring change in these over time. The production of more reliable and spatially detailed population datasets is increasingly necessary due to the importance of improving metrics at sub-national and multi-temporal scales. This is in support of measurement and monitoring of UN Sustainable Development Goals and related agendas. In response to these agendas, a method has been developed to assemble and harmonise a unique, open access, archive of geospatial datasets. Datasets are provided as global, annual time series, where pertinent at the timescale of population analyses and where data is available, for use in the construction of population distribution layers. The archive includes sub-national census-based population estimates, matched to a geospatial layer denoting administrative unit boundaries, and a number of co-registered gridded geospatial factors that correlate strongly with population presence and density. Here, we describe these harmonised datasets and their limitations, along with the production workflow. Further, we demonstrate applications of the archive by producing multi-temporal gridded population outputs for Africa and using these to derive health and development metrics. The geospatial archive is available at https://doi.org/10.5258/SOTON/WP00650.

## 1. Introduction

Human population mapping is fundamental in support of a broad range of applications by governments, non-governmental organisations, and private businesses. Detailed and up to date spatial datasets that accurately describe population distribution can support the planning and delivery of services (Langford, Higgs, Radcliffe, & White, 2008), election mapping (Amos, McDonald, and Watkins 2017), estimation of populations at risk of infectious disease or hazards (Hay, Guerra, Tatem, Atkinson, & Snow, 2005; Linard, Alegana, Noor, Snow, & Tatem, 2010; Snow, Guerra, Noor, Myint, & Hay, 2005), and disaster relief operations (Bhaduri, Bright, Coleman, & Dobson, 2002; Nadim, Kjekstad, Peduzzi, Herold, & Jaedicke, 2006; Taramelli, Melelli, Pasqui, & Sorichetta, 2010).

Census data are typically made openly available only aggregated by large administrative areas as spatial (areal) units. Aggregation results in loss of spatial detail and is performed to protect confidentiality. It is possible to directly produce human population distribution maps from such data by linking counts to the appropriate boundaries. However, the use of large spatial areal units presents analytical challenges for population studies. Administrative unit boundaries are often unrelated to the demographic variables of interest, and in the physical world populations are not uniformly distributed within them (Sorichetta et al., 2015; Stevens, Gaughan, Linard, & Tatem, 2015). Such challenges make it difficult to compare the distribution of human populations over time and space in a consistent and methodological way.

In order to better characterise the distribution of populations and overcome the limitations of such aggregate data, much research has focused on creating alternative representations of the population as a continuous surface (Mennis, 2003). Such approaches use a variety of techniques to assign estimated population counts to grid cells, a topic discussed in more detail in Wardrop et al. (2018). There are two ways to approach modelling gridded population data, either a “top-down” or a “bottom-up” approach. The top-down modelling approaches (Azar, Engstrom, Graesser, & Comenetz, 2013; Stevens et al., 2015) are the most commonly used due to the availability of census and geospatialgeospatial covariate data.

A top-down approach relies on high quality and up to date census population counts or official estimates that are combined, or “aggregated”, into administrative units and linked to their digital boundaries. Subsequently, counts are redistributed (or “disaggregated”) into grid cells (i.e. pixels). A variety of techniques may be utilised to disaggregate, ranging from the simple through to the more statistically complex. The “areal-weighting” technique is a simple way to address the challenge of characterising the spatial variation of population within administrative units, taking (non-spatial) tabular counts of population (listed by administrative unit) and (spatial) administrative boundary data, and disaggregating population from census units into grid cells through the assumption that the population of a grid cell is an exclusive function of the land area within that pixel (Doxsey-Whitfield et al., 2015). The Gridded Population of the World (GPW) v4 dataset (CIESIN, 2016a) uses the areal-weighting technique (CIESIN, 2016b), detailing population count and density at 30 arc-second resolution (approximately 1 km resolution at the equator). The advantage of this simple disaggregation technique is that it does not incorporate more complex considerations. Output grids can, therefore, be used with other geographic information without endogeneity concerns. The major disadvantage is the inability to characterise spatial variations within the input geometry, especially in cases where the input administrative units are much larger than the spatial resolution of the output grid. Dasymetric mapping is a more complex technique that uses geospatial covariates (e.g. land cover) via a spatial weighting grid to more accurately distribute the population data assigned to selected administrative units. Dasymetric mapping has been shown to be the most accurate top-down approach to disaggregating census counts into gridded maps (Sorichetta et al., 2015; Stevens et al., 2015; Wardrop et al., 2018).

In comparison, “bottom-up” approaches (Checchi, Stewart, Palmer, & Grundy, 2013; Hillson et al., 2014, 2015; Tomás, Fonseca, Almeida, Leonardi, & Pereira, 2015; Wardrop et al., 2018; Weber et al., 2018) are a more recent development that take complete counts of population within small, defined areas (sometimes called “micro-census” surveys) and produce a gridded estimate of overall population through the prediction of population in (much larger) un-surveyed areas via the use of geospatial covariates and statistical modelling (Wardrop et al., 2018). Bottom-up approaches are difficult to implement at a global scale due to the resources required to collect data, and the storage and computational overhead. Bottom-up approaches are best applied to countries where census data are of poor quality, outdated, or non-existent.

Remotely sensed and other geospatial ancillary data can be used in population modelling in order to improve detail (Balk et al., 2006; Bhaduri et al., 2002; Tatem, Noor, von Hagen, Di Gregorio, & Hay, 2007). For example, the Global Rural–Urban Mapping Project (GRUMP) version 1 (Balk, Pozzi, Yetman, Deichmann, & Nelson, 2005; CIESIN, IFPRI, World Bank, & CIAT, 2011) build on GPW v3 (CIESIN and CIAT, 2005; Balk and Yetman, 2004), differentiating urban and rural areas by formulation of a mask via the combination of census data with remote-sensed nightlights data (Balk, Yetman, & de Sherbinin, 2010; CIESIN, 2005). Land cover data may be used to redistribute aggregated census counts in order to improve the accuracy of national scale gridded population data (Linard, Gilbert, & Tatem, 2011). Where settlement extents are used, e.g. GHS-POP (Freire, MacManus, Pesaresi, Doxsey-Whitfield, & Mills, 2016), population distribution datasets are generally more accurate than when simple areal weighting is used, as shown in previous studies (Gaughan, Stevens, Linard, Jia, & Tatem, 2013; Linard et al., 2010; Linard, Gilbert, Snow, Noor, & Tatem, 2012; Linard et al., 2011; Mennis & Hultgren, 2006; Tatem et al., 2007).

A wide range of factors are known to correlate with how humans distribute themselves on the landscape (Nieves et al., 2017). A larger number of covariates may be utilised in modelling in order to more effectively disaggregate census population counts within administrative units, and to better statistically describe population distribution (Lloyd, Sorichetta, & Tatem, 2017) – an approach used to produce the Landscan population datasets (ORNL 2010; Bhaduri, Bright, Coleman, & Urban, 2007; Dobson, Bright, Coleman, Durfee, & Worley, 2000). The Random Forest-based (RF) dasymetric model, a non-parametric ensemble approach (Breiman, 2001), is a further example used to produce WorldPop population datasets (Gaughan et al., 2016; Sorichetta et al., 2015; Stevens et al., 2015). The RF method, discussed in more detail later in this paper, incorporates census data and a wide range of ancillary datasets in a flexible estimation technique. Output suggests marked improvements in mapping accuracies over other “top-down” population mapping approaches, such as areal-weighting (Sorichetta et al., 2015; Stevens et al., 2015).

Due to lack of resources (financial and human) to carry out detailed censuses, fine spatial detail population count data are lacking for the present day and past decades in many countries (e.g. Afghanistan, Democratic Republic of Congo, Lebanon, Uzbekistan, in particular), thereby limiting applications linked to specific time periods or those measuring changes. Sub-national scale analyses related to population are beginning to utilise multi-temporal geospatial layers (Bennett & Smith, 2017a, 2017b). Multi-temporal geospatial layers are useful in providing globally consistent gridded population distribution datasets that can be used to support agendas aligned with Sustainable Development Goals (SDGs) (UN General Assembly, 2015). Aligned agendas are those such as the Institute for Health Metrics and Evaluation (IHME) Global Burden of Disease (GBD) studies (GBD, 2016; IHME, 2013, 2016; SDG Collaborators, 2017) or the Malaria Atlas Project (MAP, 2017; Bhatt et al., 2015; Cibulskis et al., 2016). The present situation of a lack of multi-temporal global modelled population data limits abilities to provide context to global multi-temporal disease prevalence mapping efforts and convert them to burden estimates.

In order to better support global high-resolution population mapping in the future, a set of methods have been developed here to assemble and harmonise a unique (in spatial and temporal scope), open access, archive of geospatial datasets. Datasets are provided as annual time series, where pertinent at the timescale of population analyses and where data are available. These can be used to construct consistent and comparable annual high-resolution global population distribution layers for the 2000–2020 period. The archive includes sub-national census-based population estimates, matched to gridded administrative boundaries, and a number of co-registered gridded geospatial factors that correlate strongly with population presence and density. The datasets described in this paper are mostly an assemblage of pre-existing datasets, created to provide researchers with easier access via considerable effort towards harmonisation.

A collection of harmonised geospatial layers has previously been developed for use in population studies (Lloyd et al., 2017), as an internal effort undertaken with the WorldPop programme. The collection described here demonstrates significant differences and advancements over that earlier work and is a significant cross-organisational collaboration between WorldPop and the Center for International Earth Science Information Network (CIESIN). The pre-existing datasets used to create the geospatial layers described in Lloyd et al. (2017) are almost entirely different to those discussed in this paper and are standardised solely to less accurate Global Administrative Areas version 2 (GADMv2) (GADM, 2015) country boundaries. In contrast, the newly assembled and harmonised layers, discussed here, mark a significant improvement by the inclusion of subnational census-based population estimates and by the utilisation of associated administrative boundaries. These are the same input data as previously used in the production of the GPWv4 gridded datasets (CIESIN, 2016a, 2016b; Doxsey-Whitfield et al., 2015). Further, the geospatial layers described in Lloyd et al. (2017) are mostly time invariant, therefore, not effectively facilitating the monitoring of change in population over time, whereas the layers described in this paper are provided as time series where relevant/available.

Here, we describe the production methods for the geospatial layers. A predominantly open source production environment is utilised, and a semi-automated workflow. We then present example applications of the geospatial layers, as harmonised gridded inputs to inform an RF model to provide spatially consistent gridded population outputs (Stevens et al., 2015). In particular, we use the workflow described by Gaughan et al. (2016) to compare population outputs for Africa at several time periods and demonstrate the potential usefulness of these high spatial resolution data in health and development metric applications.

## 2. Methods

To support the production of global maps of population distributions and demographics for the period 2000 to 2020, population counts (interpolated and forecast at sub-national level) are linked to spatially and temporally harmonised national and sub-national spatial data describing administrative unit extents, derived from GPWv4 (CIESIN (Center for International Earth Science Information Network, Columbia University), 2016a). A range of open access geospatial layers are collected and similarly harmonised, representing factors that correlate strongly with human population density (Nieves et al., 2017).

**Figure 1.**
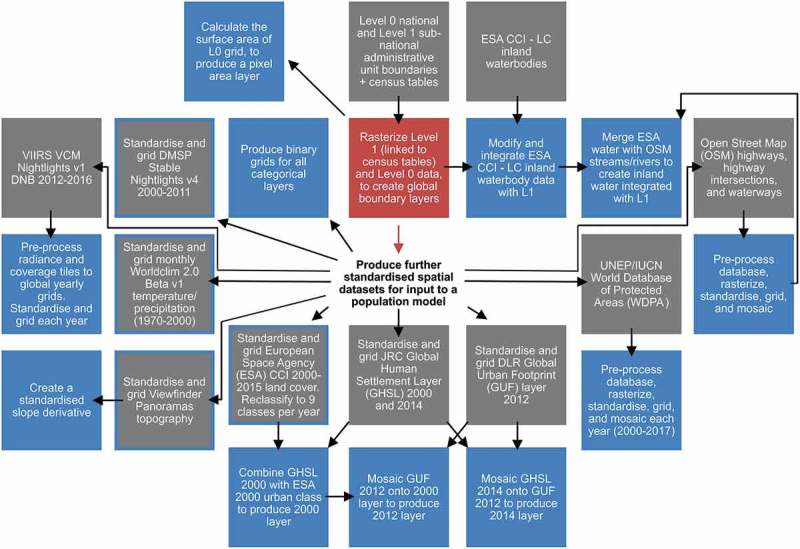
Flowchart of the workflow to produce standardised spatial datasets for potential input to a population model. Production of base datasets is depicted in red, and source data in grey. Processes which directly lead to the production of further covariate output, for potential input to a population model, are represented in blue (or blue border as appropriate).

The time-invariant geospatialgeospatial layers produced as potential input grids for modelling population distribution are: Viewfinder Panoramas (SRTM based) topography (units in metres) for year 2000 (de Ferranti, 2017a); a slope layer derived from the topography (in degrees); pixel area (m^2^), and coastline (binary, as land/open water pixels); OpenStreetMap (OSM) highway (major highway routes), highway intersection, and waterway locations (OSMF and Contributors, 2016); and WorldClim average global temperature (°C) and precipitation (mm) for 1970–2000 (Fick & Hijmans, 2017). The multi-temporal geospatialgeospatial layers (i.e. annual time series) produced are: DMSP-OLS version four night-time lights (2000–2011) composites (US NOAA, 2015; Zhang, Pandey, & Seto, 2016); VIIRS version 1 night-time lights (2012–2016) composites (US NOAA, 2017); ESA CCI annual global land cover for 2000–2015 (ESA CCI, 2017a); UNEP/IUCN World Database of Protected Areas for 2000–2017 (UNEP-WCMC and IUCN, 2017); and built settlement grids for 2000, 2012, and 2014, which combine the JRC Global Human Settlement Layer (Pesaresi et al., 2015) with the ESA CCI built settlement landcover class and the DLR Global Urban Footprint (DLR EOC, 2016) dataset and which were extrapolated and interpolated into an annual time series as described in Nieves et al. (2018). The workflow for standardising and harmonising geospatialgeospatial layers is a significant development and expansion of methods discussed in Lloyd et al. (2017), and Lloyd (2017). Workflow is visualised diagrammatically in Figure 1. Source datasets are detailed in Table 1.

**Table 1.** Source datasets used to produce geospatial raster layers for potential input to a population model.

Name	Acquisition year	Temporal variation	Source	Version, publication year	Data type	Spatial resolution	Format/ pixel type & depth	Spatial reference	Spatial coverage
National L0 and sub-national census L1 administrative boundaries	2005–2014	Time Invariant	Center for International Earth Science Information Network (CIESIN), Columbia University	GPW v4, 2016	Global population count and administrative boundaries, table and vector	Comparable to 3” (~90 m)	ESRI polygon shapefiles	GCS WGS 1984	Global
Water bodies	2000–2012	Time Invariant	ESA (European Space Agency) CCI (Climate Change Initiative) – LC (Land Cover project)	v4.0, 2017	Inland water bodies, categorical raster	4.5” (~150 m)	Geo-tiff/ uint8	GCS WGS 1984	Global
Viewfinder Panoramas Topography	~2000	Time Invariant	de Ferranti, J.	28/11/17	Elevation, continuous raster	Typically 3” (~90 m)	HGT tiles/ int16	GCS WGS 1984	Global
Open Street Map (OSM)	2016	Time Invariant	OpenStreetMap Foundation (OSMF) & Contributors	15/01/16	General mapping, categorical vector	Comparable to 1” (~30 m)	PBF database	GCS WGS 1984	Global
WorldClim 2.0	1970–2000	Time Invariant	Fick, S.E. and Hijmans, R.J.	01/06/16	Monthly temperature and precipitation, continuous rasters	30” (~900 m)	Geo-tiff/ flt32,int16	GCS WGS 1984	Global
DMSP-OLS Stable Nightlights	2000–2011	Time Series	US NOAA National Geophysical Data Center; Zhang et al.	v4, 2015; inter-calibrated, 2016	Annual night lights intensity, continuous rasters	30” (~900 m)	Geo-tiff/ uint8	GCS WGS 1984	Between latitudes 75° North and 65° South
ViiRS Cloud Mask (VCM) Nightlights Day/Night Band (DNB)	2012–2016	Time Series	US NOAA National Geophysical Data Center	v1, 2017	Monthly night lights intensity, continuous rasters	15” (~450 m)	Geo-tiff tiles/ flt32,uint8	GCS WGS 1984	Between latitudes 75° North and 65° South
ESA CCI Land Cover	2000–2015	Time Series	ESA CCI – LC	v2.0.7, 2017	Annual land cover, categorical rasters	9” (~300 m)	Geo-tiff/ uint8	GCS WGS 1984	Global
World Database of Protected Areas (WDPA)	1819–2017	Time Series	UNEP-WCMC and IUCN	June 2017	Terrestrial and marine protected areas, categorical vector	Comparable to 30” (~900 m)	ESRI geodatabase	GCS WGS 1984	Global
JRC Global Human Settlement Layer (GHSL)	2000, 2014	Time Series	Pesaresi, et al.	2015	Urban settlement, categorical rasters	1.26” (~38 m)	Geo-tiff/ uint8	Spherical Mercator projection (EPSG:3857)	~85.06 degrees North and South latitude
Global Urban Footprint (GUF)	2012	Time Invariant	DLR EOC	2016	Urban settlement, categorical raster	2.8” (~84 m)	Geo-tiff/ uint8	GCS WGS 1984	Global

Source datasets are here described. Data source, version, format, and spatial and temporal information are summarised.

Categorical covariates are further each converted to binary grids (representing the feature of interest) as additional potential input grids for modelling, and from which derivative covariates can be produced if desired. Derivatives (such as datasets that indicate the distance to a given feature) increase covariate variability and therefore better captures the relationship with population density (e.g. urban core verses outskirts).

### 2.1. Source datasets

National (L0) and sub-national (L1) administrative unit boundary vector source material (CIESIN (Center for International Earth Science Information Network, Columbia University), 2016a) are rasterised by CIESIN, forming the base grids (i.e. mastergrids) of the archive. Regarding the sub-national L1 administrative units, it is important to highlight that even though hereafter these are simply referred to as L1, they actually represent the highest administrative unit level obtained for each country. The L1 data define administrative units per country for the entire globe, and are combined by CIESIN with ESA CCI – LC v4.0 inland waterbody raster data (ESA CCI, 2017b) to form one dataset. The L0 country identification (ID) global layer uses a three digit numerical ISO 3166 country code standard (ISO, 2017) that applies country/territory names/codes as designated by the United Nations, some of which are disputed. For disputed territories the intent is not to represent international boundaries, but rather to represent the source of the census administrative unit boundary and count estimate data. The L0 layer complements the L1 data via the use of common national boundaries. Other geospatial layers are spatio-temporally harmonised (standardised) to grid definitions and coastlines derived from administrative unit extents. All source datasets use a geographical coordinate system (GCS) with WGS 1984 datum (EPSG:4326), unless otherwise detailed.

L0 and L1 administrative unit boundary source material are gridded by CIESIN to 3 arc-second (0.00083333333 decimal degree) spatial resolution. CIESIN then resample and integrate ESA CCI – LC v4.0 4.5 arc-second (~150 m at the Equator) spatial resolution inland waterbody data with L1. Administrative unit boundary data take priority where there is complete overlap of units with waterbody data. Underlap between census and water is designated as water. The chosen cell size represents a middling spatial resolution to which source datasets (having various resolutions) can be rationalised, and offers reasonable storage and computational overhead at a global scale. Such overhead would increase significantly were a finer spatial resolution chosen instead.

Sub-national population count tables are interpolated and forecast annually by CIESIN from the year 2000 to 2020, using two census dates for most countries (circa 2000, and circa 2010) taken from GPWv4. The latest population census data have been collected during the 2010 round, between 2005 and 2014 (Doxsey-Whitfield et al., 2015). Countries conduct their censuses at different times. Hence, in order to interpolate and forecast, annual growth rates are used to adjust population counts to allow for global comparison. Exponential growth rates have been calculated for each administrative unit by matching the total population from the latest census enumeration to those from a previous census enumeration. In cases where matching at the highest spatial detail is not possible between the two points in time (e.g. boundary changes), censuses have been matched and growth rates calculated at a less detailed administrative level (state/province or district), and applied to each unit (municipality) within that highest administrative level. For further detail see Doxsey-Whitfield et al. (2015) and CIESIN (2016b). The growth rate has been calculated using the following formula:

(1) $$r=\frac{ln\left(\frac{P_{2}}{P_{1}}\right)}{t}$$

where r is the annualised growth rate, P_1_ is the population count at the time of the earlier census, P_2_ is the population count from the latest census, and t is the number of years between the two. Population estimates were then calculated for the target years as follows:

(2) $$P_{x}=P_{2}e^{rt}$$

where P_x_ is the population estimate in the target year x, and P_2_, r, and t are as defined previously (CIESIN (Center for International Earth Science Information Network, Columbia University), 2016b).

National and sub-national administrative unit boundaries follow census cartography if available (CIESIN, 2018). When census cartography is not available, non-census boundaries are utilised by CIESIN if obtainable. This is in order that the full effective spatial resolution of the tabular census data may be utilised. A country is gridded at a coarser resolution only if one of the tabular census data or administrative unit boundaries are not available. Particularly for non-census boundaries, reconciliation with census data is a significant undertaking in both time and labour, discussed further in Doxsey-Whitfield et al. (2015). In order to ensure consistency between countries, administrative unit boundaries are aligned to a global framework in part based on the Global Administrative Areas version 2 (GADMv2) (GADM, 2015), sourced primarily from national governments and NGOs. GADM is utilised because it is openly available, consistent, and widely used in the research community (CIESIN (Center for International Earth Science Information Network, Columbia University), 2016b). In cases where the resolution of the administrative unit boundaries far exceed that of the GADM boundaries, the former are kept (Doxsey-Whitfield et al., 2015). Average census administrative unit resolution for highly developed regions is 936 arc-second (~31 km at the Equator), and 1764 arc-seconds (~59 km at the Equator) for less developed regions, calculated from de Sherbinin and Adamo (2015). Where country boundaries follow GADM the effective spatial resolution is comparable to that of census boundaries but varies in quality according to the original source material.

Topography data consists of the Viewfinder Panoramas dataset (de Ferranti, 2017a), which is primarily US NASA Shuttle Radar Topography Mission (SRTM) data (US NASA 2016) collected in the year 2000, has 3 arc-second (~100 m at the Equator) horizontal and 1 m vertical spatial resolution, and is amended and corrected by the dataset developer Jonathan de Ferranti (de Ferranti, 2017a). Viewfinder Panoramas data are provided filled and corrected from the best available alternative sources where SRTM data are unavailable (i.e. north of 60° 2ʹN and south of 56° S) or for some mountain and desert regions between these latitudes where there are voids and areas of phase unwrapping error (de Ferranti, 2017a, 2017b). Alternative sources are topographic maps, Landsat images, and ASTER GDEM data – sources that are much more accurate than the simple interpolation of SRTM data (de Ferranti 2017a).

OpenStreetMap (OSMF and Contributors, 2016) data (for January 2016) are global “voluntary geographic information” stored as a global database. OSM data have an effective resolution comparable with SRTM1 at 1 arc-second (~30 m at the Equator) but varies according to source data. OSM data use a system of nodes, ways, and relations to define points in space, linear features/area boundaries, and the way in which these attributes work together. Tags are used to categorise and label each attribute (OSMF, 2018a). The frequency of contributions by individual users will refine source data, as often can contributions from out of copyright maps (OSMF, 2018b) or contributions from professional cartographic organisations (OSMF, 2018c).

The WorldClim 2.0 Beta version 1 (Fick & Hijmans, 2017) global temperature (°C) and precipitation (mm) data are each provided as 12 30 arc-second (~1 km at the Equator) spatial resolution raster images representing average monthly climate data for the period 1970–2000.

DMSP-OLS version 4 stable night-time lights (2000–2011) annual composite time series (US NOAA, 2015) are light intensity data provided as raster layers with near global coverage between latitudes 75 degrees North and 65 degrees South. Source data have 30 arc-second (~1 km at the Equator) spatial resolution. For the years 2000–2007 (inclusive) data are available from two satellites, whereas for the years 2008–2011 (inclusive) data are available from one satellite. The stable composite product contains lights from cities, towns, and other sites with persistent lighting, including gas flares. Ephemeral events, such as fires are not included (US NOAA, 2015). An inter-calibrated version of the stable night-time lights annual composites (Zhang et al., 2016), which provides relative radiometric calibration and saturation correction is utilised to produce the global harmonised lights data for this study up to the year 2011. The unit of radiance employed in the standard uncalibrated DMSP data is a digital number ranging from 0 to 63. The inter-calibrated version of the data multiplies the digital number of the source by 100.

Similarly, for 2012 to 2016 (inclusive) we use VIIRS Cloud Mask (VCM) version 1 night-time lights Day/Night Band (DNB) monthly composite time series (US NOAA, 2017) light intensity data, which is provided as inter-calibrated tiled raster layers with near global coverage between latitudes 75 degrees North and 65 degrees South. Source data have 15 arc-second (~450 m at the Equator) spatial resolution. Twelve monthly average radiance composites are available for each year 2013–2016, and 9 for 2012 (April–December inclusive). Each monthly composite is divided into six tiles (75°N, 180°W; 75°N, 60°W; 75°N, 60°E; 0°N, 180°W; 0°N, 60°W; 0°N, 60°E). For each tile and each month, there is also a cloud-free observation raster that records how many cloud-free observations have been made by the satellite for each pixel within the average radiance image. These coverage files allow the end user to differentiate between no data pixels (i.e. in this case zero observations due to cloud cover) and pixels where observations were made but no lights were observed. The DNB VCM version of the data excludes data impacted by stray light, lightning, lunar illumination, and cloud cover. Version 1 data are not filtered to screen out lights from aurora, fires, boats, and other temporal lights (US NOAA, 2017). The unit of radiance employed by the VIIRS DNB data is nanoWatts/cm2/sr. Original radiance values are multiplied by 1E9 in the source data.

ESA CCI annual global land cover time series (2000–2015) version 2.0.7 (ESA (European Space Agency) CCI (Climate Change Initiative) – Land Cover project 2017, 2017a) classifies land use sub-categories for agriculture, forest, grassland, wetland, settlement, and other (including water) (ESA CCI, 2017c). Source data are provided as raster layers with global coverage, and a 9 arc-second (~300 m at the Equator) spatial resolution.

United Nations Environment Programme World Conservation Monitoring Centre (UNEP-WCMC) World Database of Protected Areas (WDPA), version June 2017 (UNEP-WCMC and IUCN, 2017) is the most comprehensive global database on terrestrial and marine protected areas (Chape, Harrison, Spalding, & Lysenko, 2005), comprising both spatial data (i.e. boundaries) and attribute data (i.e. descriptive information) for all protected areas from 1819 to 2017 (UNEP-WCMC, 2017). The International Union for Conservation of Nature (IUCN) Protected Area Management Categories, stored within the database, help classify protected areas based on their primary management objectives (Dudley, 2008). Effective resolution varies according to original source data (UNEP-WCMC, 2017; Visconti et al., 2013).

JRC Global Human Settlement Layer (GHSL) (Pesaresi et al., 2015) GHS BUILT LDS2000, and LDS2014, GLOBE R2016A 3857 38 grids detail built-up presence of settlement for years 2000 and 2014 respectively. Data are provided per year, each split into two rasters with cumulative near global coverage (~85.06 degrees North and South latitude), at 1.26 arc-second (~38 m at the Equator) spatial resolution, in Spherical Mercator projection (EPSG:3857) (GHSL, 2015).

The DLR Global Urban Footprint (GUF) (DLR EOC 2016) GUF28 v1 raster grid details the built-up presence of settlement for the year 2012. Data are provided with global coverage, and 2.8 arc-second (~84 m at the Equator) spatial resolution (Esch et al., 2017).

### 2.2. Production of datasets

The methods used to harmonise the datasets are here described. We subsequently use produced geospatial layers as input to an RF model (Stevens et al., 2015), using methods for temporal considerations described by Gaughan et al. (2016) to produce gridded population outputs and demonstrate applications for such data.

#### 2.2.1. Processing software

Open source OSGEO4W64 geospatial Software (OSGF, 2017a) and the included geospatial Data Abstraction Library (GDAL) v2.1.3 package (OSGF, 2017b) are employed to produce archive datasets, using a Microsoft Windows 7, 64-bit operating system (OS). Occasionally proprietary ESRI ArcMap v10.3.1 and ArcInfo Workstation v9.3 GIS software (ESRI, 2016) are utilised where specific functionality is otherwise unavailable. Program code is implemented as windows batch script files within OSGEO4W64 at command line unless otherwise stated. Scripts and supporting “readme” files are available to download from Figshare (Lloyd, Chamberlain, Kerr, & Bondarenko, 2018). ESRI ArcMap v10.3.1 is employed to create the Level 0 and Level 1 tiled data. Python (v.3.6) (PSF, 2016) and the included Pandas module is used to interpolate the time series of population data, and to merge missing records.

Software used for initial OSM database processing is as described in Lloyd et al. (2017). Subsequent database access, filtering, and processing (on the Windows platform) are provided by QGIS 2.18.4 (QGIS project, 2017) and Spatialite v4.3.0a, including the Spatalite graphical user interface (GUI) 2.0.0 (Furieri, 2016) software. GDAL, and SAGA GIS 4.1.0 (SAGA 2017) command line utilities are used to convert to raster format and standardise the data. The processing of the UNEP/IUCN WDPA utilises PostgreSQL 9.1 (PostgreSQL GDG, 2016) and PostGIS 2.0 (PostGIS PSC, 2016) database software, and GDAL. The standardisation of output has utilised the IRIDIS 4 High-Performance Computing (HPC) Facility at the University of Southampton, using a Linux OS (Redhat 6) and GDAL version 1.10.1. Similarly, GHSL rasters are processed using GDAL on the HPC, owing to source spatial resolution and the associated computational overhead.

#### 2.2.2. Viewfinder panoramas topography, and slope derivative

All Viewfinder Panoramas topography tiles are first mosaicked into one global image using GDAL utilities. The topography data is standardised to grid definition and coastlines. No data pixels at coastal edges (present due to inconsistencies in coastline location between topography and L0 data) are filled, to produce the global topography layer. A global slope layer is created from the topography data using GDAL.

#### 2.2.3. L0/L1 derivatives

To create the pixel area grid, an ARC Macro Language (AML) script (modified from Santini, Taramelli, & Sorichetta, 2010) calculates the surface area of cells in a regularly spaced longitude-latitude (geographic) grid of the Earth’s surface at 60 arc-second resolution, using ESRI ArcInfo (Arc) software. Our approach to the surface area calculation is based on the spherical approximation of the Earth’s surface described by Santini et al. (2010). The production workflow is a refinement of that described in Lloyd et al. (2017). A binary grid of coastline is created from the L0 country data. Binary grids are created for all produced categorical covariates, for potential application in modelling.

ESA CCI – LC v4.0 inland waterbody data (modified by CIESIN) are extracted from the L1 data and mosaicked onto OSM “waterway” tagged polylines (streams and rivers) to provide a contiguous inland water dataset that is fully integrated with the L1 census unit data. A separate contiguous OSM inland water (streams, rivers, lakes, etc.) layer is also created as an alternative dataset.

#### 2.2.4. OpenStreetMap (OSM)

After initial OSM database processing, relevant data are exported and converted into raster format. In common with the workflow of Lloyd et al. (2017), QGIS is utilised to modify each database table (i.e. point, line, and polygon) and to convert the database attributes of interest into spatialite tables (i.e. spatially enabled SQLite databases) in order to allow greater and faster manipulation of spatial data than would otherwise be possible if working directly with the source database. A classification field is added to each spatialite table if required in order to rank features (such as the priority of roads in the highway network) for later preservation as pixel values when tables are converted to raster format (e.g. higher priority roads take precedence). Assignment simplifies tagging so as to be manageable for display in raster format. Subsequently, attribute (tag) extraction from a given spatialite table, further processing specific to each subset (i.e. highways, waterways, etc.), and conversion to raster format can take place, using a combination of QGIS, GDAL (ogr2ogr utility, using sqlite SQL dialect) and SAGA GIS. For each subset, the relevant tagging filters utilised during extraction, any associated variants and/or misspellings of tags, as well as excluded tags, are detailed in the supplementary code. Particular attention has been paid to manual examination of OSM tagging in order to extract maximum information from OSM data across all subsets. For reasons of computational efficiency during the execution of certain algorithms (e.g. intersection), spatialite tables are tiled for processing before rasterisation and standardisation. Further, specific workflow for each OSM subset are here summarised, with further detail supplied in the supplementary material.

#### 2.2.5. OSM highways

A highways layer with “highway” tags assigned a classification field “priority” value of 1–17 (footpath to motorway road classes) is created. Priority value assignment is detailed in Supplementary Table 1. A “bridge” and “tunnel” tagged (henceforth referred to as “links”) layer is also created, only for those “inter-coast” highways situated over/under water (e.g. bridges and tunnels at estuaries, narrow sea ways, etc.). Such links are removed during the standardisation of highway rasters. Creation of the layer allows links to be restored after standardisation, so that coastal roads remain contiguous. Links are given an arbitrary priority value (of 30) to differentiate them from the rest of the road network. This part of the workflow follows that of Lloyd et al. (2017), albeit subsequently standardising to more accurate coastal boundaries.

Highway and links sets of spatialite tiles are each separately converted to a vector format using GDAL (ogr2ogr) for compatibility with the SAGA GIS rasterisation (“Shapes to grid”) command line tool. Using GDAL, two copies of L0 raster tiles (at 100 m spatial resolution) are made, with country code values reset to an arbitrary value, and tile extents identical to the vector tiles. Onto these copies are rasterised the maximum priority attribute value that is apparent per 100 m pixel, for each set. Each set of raster tiles is mosaicked, and the highway mosaic standardised. The links mosaic is standardised so that only those features located offshore are retained. The two standardised layers are mosaicked together and background land values set to zero. A calculation is performed to produce the final highways layer for classes 8–30 (i.e., tertiary to motorway road classes, plus links). In addition, highway priority classes are each extracted individually (for classes 8–30) and rasterised separately using the same method in order to increase variability during modelling. Highway classes 8–30 are considered to be major highway routes, which are particularly well correlated with population density and so are significant for the purpose of modelling. Lesser classes are excluded.

#### 2.2.6. OSM highway intersections

A highway intersection layer for road priorities of 8–17 (tertiary to motorway road classes) is created. These classes are selected because they represent only major highway routes and therefore provide only “significant” highway intersections. The inclusion of lower classes of the road via (for example) introduction of residential streets (class 7) into the layer would provide overly dense and potentially misleading intersection information after rasterisation (particularly in urban centres). Exclusion of higher classes would remove otherwise useful intersection information. Road intersection points are rasterised using the same process as described for highways (but using the simpler data/no data output value option). Prior processing to identify intersection uses the highway spatialite table and utilises ogr2ogr and associated PostGIS functions (PostGIS PSC, 2017a, 2017b).

Our approach to identifying intersection defines highways as having uniform road name, reference number, junction, and priority tags. Where one of these criteria change, an intersection will be found. Our approach identifies where highways cross bridges and tunnels. Geospatial utilities will identify intersections at such crossing points, where of course no such highway intersections exist in real life. Such false intersections are entirely removed by our technique. Further technical elucidation regarding the intersection method can be found in the supplementary material.

#### 2.2.7. OSM waterways

A natural waterway layer is created. Three types of natural water attributes are separately extracted from the database. These attributes are “waterway” polylines (streams and rivers), riverbank polygons (where rivers, or similar, have a quantifiable width at source data resolution), and lake polygons (or similar). Canal waterways are included, despite being anthropogenic, because of their relevance to human population, transportation, and water supply. Filtered attributes are converted to three spatialite tables, extracted, rasterised, and standardised. Waterbodies are mosaicked onto riverbanks, and in turn onto waterways, using GDAL, to form one contiguous water layer.

#### 2.2.8. Worldclim 2.0 beta version 1

##### 2.2.8.1. Temperature

To create an average annual global temperature layer for the period 1970–2000, the 12 average monthly temperature rasters for the period are averaged using ESRI ArcMap Raster Calculator tool (ESRI, 2018a). The output raster is partially standardised (i.e. only spatial alignment, resolution, no data value) to a 1 km resample and reclassification (to “zero” value, ocean no data) of our coastline grid, using GDAL. In order to fill no data pixels at coastal edges, no data values in the partially standardised grid are modified to zero, converted to actual values, and output is summed with the 1 km coastline grid. Coastal areas in the modified grid are “nibbled” using the ESRI ArcMap Nibble tool (ESRI, 2018b), using the original partially standardised raster as a mask. Only data values are allowed to nibble into areas defined in the mask raster (ESRI, 2018b). Prior to use of the nibble tool, the values of each input grid are multiplied by one million (in order to preserve data precision), and then each grid is converted to integer format as a requirement of the tool. Output from the nibble tool is converted back to float format and the previous multiplication calculation reversed in order to restore original values. Output is then resampled to 100 m resolution using bilinear interpolation in GDAL and standardised to L0 coastlines. An ocean mask is applied and no data values asserted.

##### 2.2.8.2. Precipitation

To create an average annual global precipitation layer for the period 1970–2000, the 12 average monthly precipitation rasters for the period are summed using GDAL. The output raster is partially standardised (i.e. only spatial alignment, resolution, no data value, data type) and then the same workflow as described for the Worldclim temperature grid is followed (where applicable).

#### 2.2.9. DMSP-OLS version 4 stable night-time lights (2000-2011) annual composite time series

A time series of near global night-time lights annual composites is created for 2000–2011 using GDAL. Inter-calibrated annual composite input radiance rasters are averaged where data is available from two satellites (i.e., 2000–2007). The eight output grids and the grids representing 2008–2011 are subsequently standardised, being resampled to 100 m spatial resolution using nearest neighbour technique. Areas of no data coverage in polar regions are replaced with zero values, an ocean mask is applied, and the no data value asserted for each grid.

#### 2.2.10. VIIRS cloud mask (VCM) version 1 night-time lights (2012-2016) Day/Night Band (DNB) annual composite time series

A time series of near global night-time lights annual composites is created for 2012–2016 using GDAL. For a specified input raster tile and year, annual average nightlights radiance values are calculated. Values in the 12 monthly average radiance input rasters per year are summed (or nine in the case of 2012). The equivalent “cloud-free observations” coverage rasters are converted to binary (to reflect which pixels have cloud-free observations and which have none, in any given month) and summed in order to identify no data pixels for each year. Using the output, a calculation is performed to eliminate from the summed radiance tiles, pixels with no recorded observations – and to attenuate summed radiance pixel values by the number of months for which night lights have been observed (rather than by the cumulative number of observations per year – monthly radiance input rasters are already averaged per month). By this method, tiles are created that display average radiance for each year. Radiance and coverage tiles are mosaicked per year, and a calculation performed on each annual radiance mosaic – utilising the annual coverages as masks in order to interpolate no data pixels using surrounding values. Output grids are then standardised as for DMSP night lights grids.

#### 2.2.11. ESA CCI annual global land cover time series (2000-2015)

In order to create an annual global land cover time series for 2000–2015, land use sub-category classifications are extracted and simplified (to nine classes) for each annual input grid, for the efficiency of use in population analyses. GDAL is used throughout. The aggregated reclassifications can be found in Supplementary Table 2.

The output grids (containing individual aggregated classes) are summed to produce one raster (containing all aggregated classes) for each year and standardised as for the night lights grids (where applicable). Classes are individually extracted from the standardised grid for each year and converted to binary (1,0, no data) and single value (1, no data) stand-alone layers. Production of binary classes and simplification of the land cover grid increases variability for potential use in modelling. Built settlement datasets (or equivalent land cover classes) are particularly well correlated with population density and so are significant for the purpose of modelling (Nieves et al., 2017).

#### 2.2.12. UNEP/IUCN world database of protected areas (WDPA)

To create an annual global time series, detailing the extent of (terrestrial/coastal/marine) protected areas from 2000–2017, source geodatabase polygons are dissolved based on year of designation and level of IUCN protection category. Some protected areas are represented by points in the database. Points are buffered by 70 m and the resulting circles used as a proxy for protected areas. This pre-processing has been undertaken by CIESIN. The remainder of processing is undertaken using GDAL. The database is imported into PostGIS using ogr2ogr. Geometry errors triggered by differing table rules (between ESRI and PostgreSQL) are rectified using the PostGIS command ST_MakeValid (PostGIS PSC, 2017a). Two integer classification columns are added to the table in order to facilitate SQL queries. One duplicates the marine code (0 = terrestrial; 1 = coastal; 2 = marine), and the other the ICUN protection category (1 = ICUN 1a and 1b; 0 = other categories).

Polygons are rasterised (using gdal_rasterise) incrementally on an annual basis from 2000–2017. The 2000 grid includes all prior years. For computational efficiency, years prior to 2000 are rasterised decadally up to 1960, and then annually until 2000 – and mosaicked. Subsequent years are each incrementally mosaicked onto the previous year. This process leads to four rasters being produced for each year from 2000–2017 – an ICUN category ‘1ʹ and an ICUN category “others” raster for each of terrestrial and marine/coastal protected areas. In total, 72 output rasters, therefore, represent the 18 years. Subsequent processing is undertaken using the HPC. The 36 Terrestrial rasters are standardised and mosaicked onto the marine counterparts. Marine rasters are partially standardised (i.e. not to coastlines) as coastal protected areas straddle marine and terrestrial environments. The result is 36 protected areas rasters, two per year – each denoting an ICUN category.

#### 2.2.13. JRC global human settlement layer (GHSL) & DLR global urban footprint composites for 2000, 2012, and 2014

In order to create a built settlement time series (2000, 2012, 2014), which can subsequently be extrapolated and interpolated annually as per work by Nieves et al. (2018), the GHSL built settlement grids for years 2000 and 2014 are produced. Each year is provided as two rasters. The two rasters are first joined and re-projected from Spherical Mercator projection (EPSG:3857) to geographical coordinate system (GCS) with WGS 1984 datum (EPSG:4326). Each yearly grid is then standardised, being resampled using nearest neighbour technique. Areas of no data coverage in polar regions are replaced with zero values, the value used to denote built settlement modified, and the no data value asserted for each grid. GUF built settlement data for the year 2012 are provided as a single raster. This grid is standardised as for GHSL (where applicable).

For the purpose of refining the accuracy of the built settlement grids, the GHSL 2000 layer is combined with the ESA CCI 2000 built landcover class. An ESA 2000 settlement pixel is only retained in the final year 2000 layer if further classified as a settlement pixel in GUF 2012. The 2012 and 2014 built settlement layers are, respectively, created by mosaicking GUF 2012 onto the year 2000 layer, and by mosaicking GHSL 2014 onto GUF 2012. The refined multi-temporal built settlement outputs are particularly well correlated with population density and so are significant for the purpose of modelling. The rationale for combining layers is that GHSL has large areas of densely built settlements that are missing due to imagery or atmospheric conditions at the time of collection. The ESA built settlement class is back-filtered using GUF (more accurate radar data) because the ESA data is more likely to have errors of commission due to roads, bare soil, etc. (owing to the nature of the satellite sensor). In back-filtering, a limit is placed on where ESA data is allowed to fill gaps in the GHSL.

## 3. Technical validation

Harmonised datasets produced for this paper have been obtained by processing input source data to produce consistent 3 arc-second outputs. Source data are validated by independent studies (Brigham, Gilbert, & Xu, 2011; Cao & Bai, 2014; CIESIN 2016c; ESA, 2017c; Esch et al., 2017; Fick & Hijmans, 2017; Henderson, Yeh, Gong, Elvidge, & Baugh, 2003; Hormann, 2018; Iwao, Nishida, Kinoshita, & Yamagata, 2006; Lloyd et al., 2017; Min, Gaba, Sarr, & Agalassou, 2013; Muck, Klotz, & Taubenbock, 2017; Pesaresi et al., 2016; Rabus, Eineder, Roth, & Bamler, 2003; Rodríguez et al., 2005; UNEP-WCMC, 2017; US NOAA, 2017; Varga & Bašić, 2015; Visconti et al., 2013). An exception is Open Street Map source data, which do not comply with standard quality assurance procedures (Haklay, Basiouka, Antoniou, & Ather, 2013) because OSM is “volunteered geographical information” provided by any number of individual contributors. However, OSM data have intrinsic quality assurance through analysis of the number of contributions for a given spatial unit. The assumption that as the number of contributors increase then so does the quality of the data is known as “Linus” Law’. Recent studies show that for OSM data this rule applies with regard to positional accuracy (Haklay et al., 2013). Whilst effective spatial resolution of OSM data is high, there is a lack of sufficiently standardised user tagging of attributes. This can cause inaccuracies and difficulties in map rendition (Lloyd et al., 2017). We provide the harmonised OSM data as a time-invariant layer in order to minimise issues common in volunteered geographic information relating to data completeness and heterogeneity. As of January 2016, OSM highway data are estimated to be globally ~83% complete with more than 40% of countries, including several in the developing world, having a fully mapped street network (Barrington-Leigh & Millard-Ball, 2017). Only the most significant road classes are included in the harmonised output, as these are likely the most complete globally. A further exception in terms of quality assurance is WDPA source data, which are subject to a series of quality checks and reformatting (by WDPA) to ensure that data standards are met. However, due to the inherent variability of data submitted by a wide range of providers with different capacity and resources to digitise protected area boundaries, issues with the accuracy of the WDPA should be expected (UNEP-WCMC, 2017). Discrepancies generated by such differences in resolution are discussed in Visconti et al. (2013).

## 4. Dataset value

**Figure 2.**
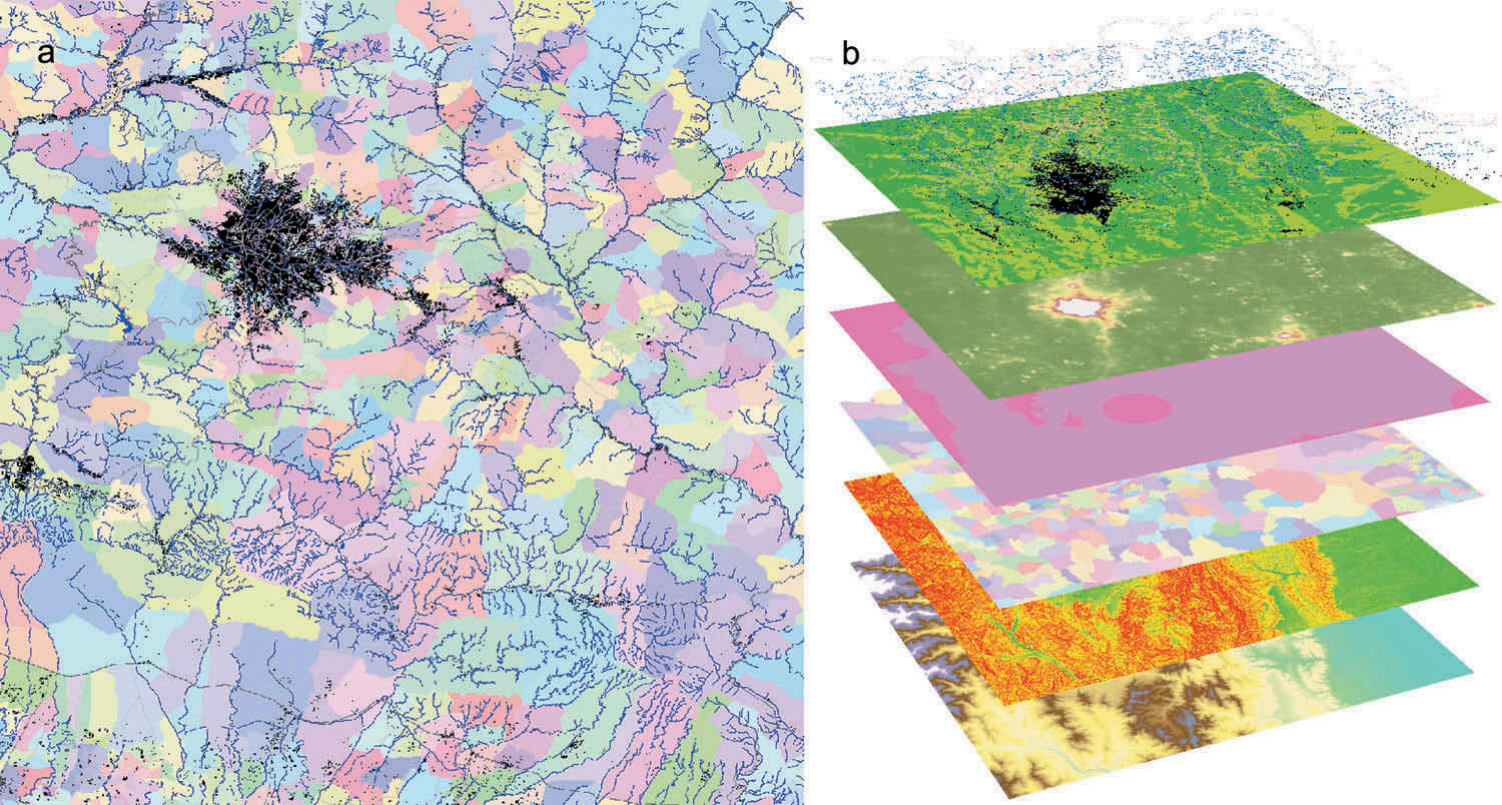
Geospatial raster layers produced for potential input to a population model.

The archive of harmonised geospatial layers is summarised in Table 2, with a visualised sample presented in Figure 2. Further, we present applications of the geospatial layers, as input to a Random Forest model (Gaughan et al., 2016; Stevens et al., 2015), and demonstrate the potential usefulness of these data in health and development metric applications.

**Table 2.** Geospatial raster layers produced for potential input to a population model.

Name	Acquisition year	Temporal variation	Source	Version, publication year	Data type	Spatial resolution	Format/ pixel type & depth	Spatial reference	Spatial coverage
National L0 and sub-national census L1 administrative boundaries with integrated waterbodies	2005–2014/ 2000–2012	Time Invariant	Center for International Earth Science Information Network (CIESIN), Columbia University/ ESA CCI – LC	GPW v4, 2016/ v4.0 2017	Global population count and administrative boundaries, inland water bodies, table and categorical rasters	3” (~90 m)	Geo-tiff/ uint16,uint32	GCS WGS 1984	Global
Pixel area	Derived from calculated Earth surface area grid and the country ID L0 layer				Pixel area, categorical rasters	3” (~90 m)	Geo-tiff/ uint32	GCS WGS 1984	Global
Topography	~2000	Time Invariant	de Ferranti, J.	28/11/17	Elevation, continuous raster	3” (~90 m)	Geo-tiff/ int16	GCS WGS 1984	Global
Slope	Derived from topography				Slope, continuous raster	3” (~90 m)	Geo-tiff/ uint8	GCS WGS 1984	Global
Open Street Map (OSM)	2016	Time Invariant	OpenStreetMap Foundation (OSMF) & Contributors	15/01/16	Highways, highway intersections, waterways, categorical rasters	3” (~90 m)	Geo-tiff/ uint8	GCS WGS 1984	Global
WorldClim 2.0	1970–2000	Time Invariant	Fick, S.E. and Hijmans, R.J.	01/06/16	Annual temperature and precipitation, continuous rasters	3” (~90 m)	Geo-tiff/ flt32, flt32	GCS WGS 1984	Global
DMSP-OLS Stable Nightlights	2000–2011	Time Series	US NOAA National Geophysical Data Center; Zhang et al.	v4, 2015; inter-calibrated, 2016	Annual night lights intensity, continuous rasters	3” (~90 m)	Geo-tiff/ int16	GCS WGS 1984	Between latitudes 75° North and 65° South
ViiRS Cloud Mask (VCM) Nightlights Day/Night Band (DNB)	2012–2016	Time Series	US NOAA National Geophysical Data Center	v1, 2017	Annual night lights intensity, continuous rasters	3” (~90 m)	Geo-tiff/ flt32	GCS WGS 1984	Between latitudes 75° North and 65° South
ESA CCI Land Cover	2000–2015	Time Series	ESA CCI – LC	v2.0.7, 2017	Annual land cover, categorical rasters	3” (~90 m)	Geo-tiff/ uint8	GCS WGS 1984	Global
World Database of Protected Areas (WDPA)	2000–2017	Time Series	UNEP-WCMC and IUCN	June 2017	Terrestrial and marine protected areas, categorical rasters	3” (~90 m)	Geo-tiff/ uint8	GCS WGS 1984	Global
Urban Settlement	2000, 2012, 2014	Time Series	ESA CCI – LC / Pesaresi, et al. / DLR EOC	2017/ 2015/ 2016	Urban settlement, categorical rasters	3” (~90 m)	Geo-tiff/ uint8	GCS WGS 1984	Global
Binary grids, for all categorical layers	-	-	-	-	Presence of features, categorical rasters	3” (~90 m)	Geo-tiff/ uint8	GCS WGS 1984	Global

Potential population model input datasets are here described. Data source, version, format, and spatial and temporal information are summarised. See Methods section for production workflow.

A sample of harmonised gridded layers is visualised. (A) shows a plan view of L1 sub-national administrative boundaries for the region surrounding the city of Hetauda, situated in the Makwanpur District of the Narayani Zone of southern Nepal, superimposed with harmonised 2014 built settlement (symbolised in black), and 2016 OSM roads (grey), and waterways (blue). (B) shows a pseudo-3D stack of grids for the same location, with (in ascending order) topography (ascending, blue-white), slope (green-red), L1 administrative units, protected conservation areas (2016), ViiRS nightlights (2016; green-white), ESA CCI reclassified land cover (2015; vegetation – green shades; waterbodies – blue; built settlement – black), built settlement (2014; black), and OSM layers (roads – orange; waterways – blue).

### 4.1. Random forest model

An RF-based dasymetric modelling approach is utilised to produce initial population count outputs. The approach is described in Stevens et al. (2015). We utilise the model to incorporate census data and a combination of the open access, remote-sensed and geospatial datasets discussed in this paper, in order to contribute to modelled dasymetric weights (Stevens et al., 2015). The RF model is used to generate a gridded prediction of population density at 3 arc-second spatial resolution (approximately 90 m resolution at the equator). This prediction layer is utilised as the weighting surface to perform dasymetric redistribution of census population counts to the pixel level all across a country in order to obtain the population distributions at a scale finer than the source subnational administrative units (Stevens et al., 2015).

### 4.2. Application 1: change in population at risk of p.falciparum malaria in Africa between 2000 and 2014

**Figure 3.**
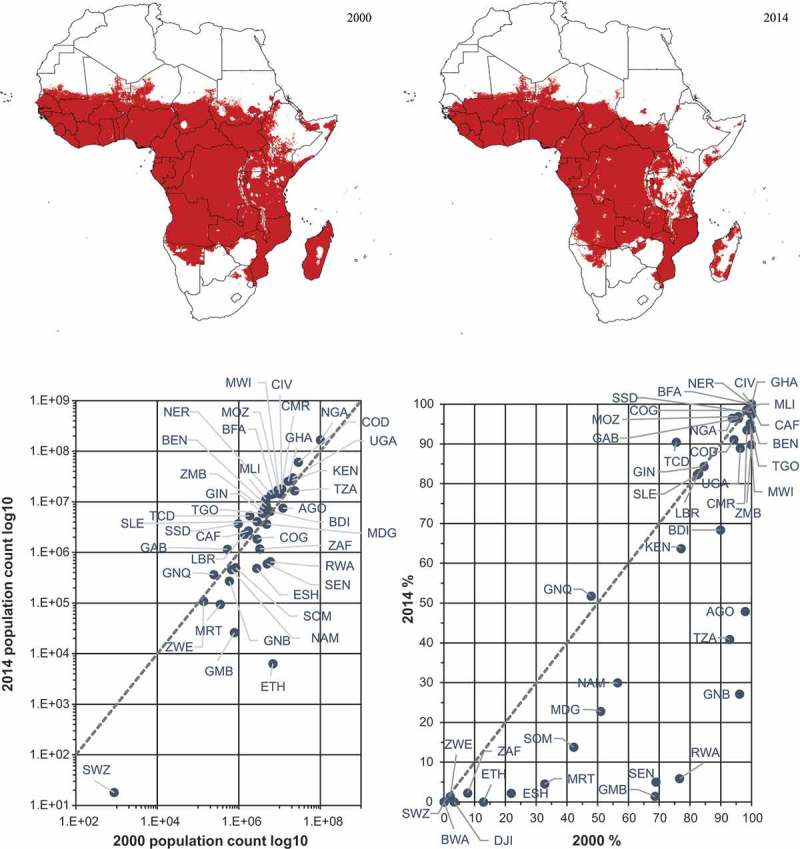
(Top) *Plasmodium falciparum* malaria prevalence rate, where >10%, for the years 2000 and 2014. (Bottom Left) Log10 of change in country population (count) at risk of *Plasmodium falciparum* malaria infection between 2000 and 2014, where prevalence is >10%. Countries (identified by ISO 3166 standard) below the trend line demonstrate a decrease in actual population count at risk of malaria infection between the respective years. (Bottom Right) Change in percentage of country population at risk of *Plasmodium falciparum* malaria infection between 2000 and 2014, where prevalence is >10%. Countries below the trend line demonstrate a decrease in the percentage of the total country population at risk of malaria infection between the respective years.

The Malaria Atlas Project (MAP) has used gridded population data as the denominator in malaria prevalence calculations for many years. It is this data and associated graphical output that is used in the (WHO World Malaria Report, 2015), produced annually. However, the MAP rely on a static denominator and some basic interpolation assumptions (Bhatt et al., 2015). We use the MAP modelled parasite prevalence rate of Plasmodium falciparum malaria (i.e. the proportion of the population with detectable parasites per year; Figure 3, Top) (Bhatt et al., 2015), as well as initial population count outputs from the previously published RF model (Stevens et al., 2015), in order to present the Log10 of change in country population (count) and the change in percentage of country population (Figure 3), at risk of Plasmodium falciparum malaria infection between 2000 and 2014 where prevalence is >10%. The table output can be found in Supplementary Table 3.

One of the Millennium Development Goals (MDGs) aimed to halt and begin to reverse the spread of malaria by 2015 (UN General Assembly, 2000). This target has been achieved – between 2000 and 2015, new cases in Africa fell by 42%, with mortality rates falling by 66% (WHO, 2015). However, progress has since stalled (WHO, 2017). Our output shows that, in many instances, country population at risk of malaria has fallen drastically between 2000 and 2014. Whilst a significant reduction in prevalence (where >10%) is apparent when the MAP data for the two time periods are compared (Figure 3, Top), the powerful combination of multi-temporal population data and malaria data for the same periods facilitates a very clear and detailed graphical (Figure 3, Bottom) and tabular (Supplementary Table 3) representation (and, therefore, understanding) of the change in actual country population count, and change in percentage of country population, at risk. It is clear that particularly good progress in risk reduction has been made in Gambia (a 68% reduction), Rwanda (71%), Senegal (64%), Guinea-Bissau (69%), Tanzania (52%), and Angola (50%), to name a few – but that there is still much work to do, with little to no progress made since 2000 in many other countries such as Ghana (in which nearly 100% of the population is still at risk), Mali (the same), Malawi (a 5% reduction, to nearly 95% risk), Mozambique (a 1% increase in risk since 2000, to 97%), and Nigeria (a 2% increase, to 96%). By using multi-temporal population data we can uncover trends about how in some countries the proportion and numbers at risk are increasing, despite general prevalence declines.

### 4.3. Application 2: change in population living in proximity to conflict in Africa between 2000 and 2014

**Figure 4.**
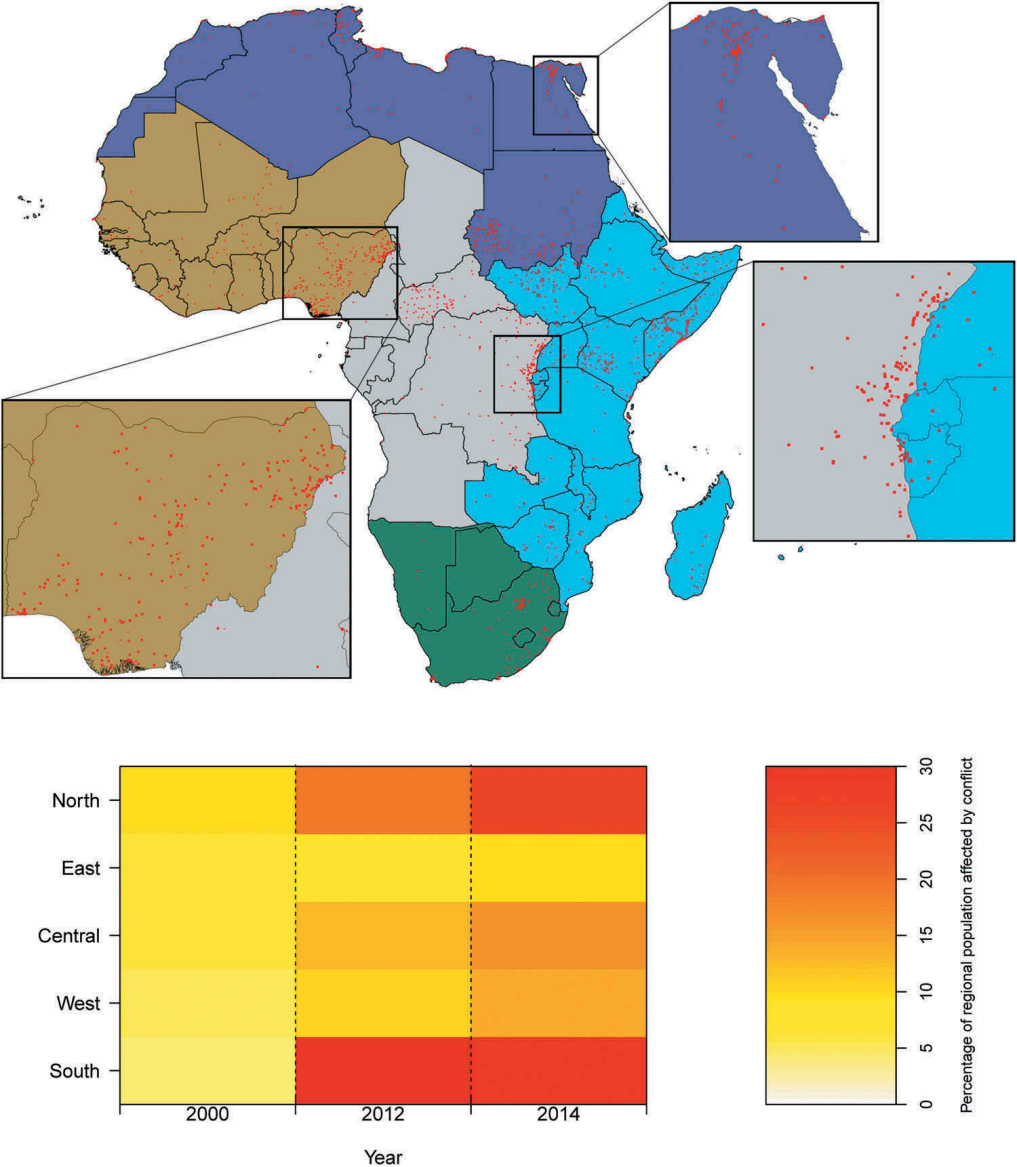
(Top) Zones (red dots) containing two or more conflict events in 2014, per each African region (Northern, Eastern, Central, Western, and Southern; depicted in purple, blue, grey, olive, and green, respectively). Break-out boxes show the same for Nigeria; The Nile, Egypt; and the eastern border of the Democratic Republic of the Congo. (Bottom) Change in the percentage of the regional population living in proximity to conflict, between 2000, 2012, and 2014, per each African region.

Understanding the numbers impacted by conflict, and associated displacement trends, can be important for humanitarian relief contingency planning, as well as long term government policy. Conflicts are very geographically focussed and fluctuate a lot over time. Hence, there is a need for spatially detailed multi-temporal population data to obtain these metrics. We use the Armed Conflict Location & Event Data Project (ACLED (Armed Conflict Location & Event Data Project), 2018) disaggregated conflict and crisis mapping for Africa for years 2000, 2012 and 2014, and corresponding initial population count outputs from the previously published RF model (Stevens et al., 2015), to present the change in percentage of population living in proximity to conflict for each African region (North, East, Central, West, South) as defined by the UN Department of Economic and Social Affairs (2018). For each region and year, populations are considered to be proximal to a conflict where within a 9 × 9 km zone containing two or more conflict events. For the purpose of this example application of the multi-temporal data, zone size has been selected to represent a reasonable area within which people may be displaced as a result of a conflict event. The zones are displayed in Figure 4 (Top), per each region. Figure 4 (Bottom) depicts the percentage change over time of regional population living in proximity to conflict, per each region. The table output can be found in Supplementary Table 4. ACLED collects the dates, actors, types of violence, locations, and fatalities of all reported political violence and protest events across Africa, as well as elsewhere. Political violence and protest include events that occur within civil wars and periods of instability, public protest and regime breakdown (ACLED (Armed Conflict Location & Event Data Project), 2018).

MDGs did not specifically mention conflict (e.g. civil wars, inter-state wars, and violence against civilians). A downward trend in the annual frequency of conflict in the world ended in the mid-2000s. Of 55 conflict-affected countries in 2015, 37 (67%) had met only two or fewer of the 15 MDGs (Norris, Dunning, & Malknecht, 2015). At least 20 of these countries are African (Themnér & Wallensteen, 2012). Even within otherwise stable countries, conflict-affected areas fared worse than areas with less or no conflict (MPSMRM (Ministère du Plan et Suivi de la Mise en œuvre de la Révolution de la Modernité), MSP (Ministère de la Santé Publique), and ICF International, 2014). Our output demonstrates that the change in the population living in proximity to conflict, between 2000, 2012, and 2014, is in line with the accepted consensus that the conflict situation has deteriorated during this period. As is the case in the malaria application, the powerful combination of multi-temporal population data and conflict zone data (Figure 4, Top) facilitates a very clear/detailed graphical (Figure 4, Bottom) and tabular (Supplementary Table 4) representation/understanding of the change in the percentage of population living in proximity to conflict. It is clear that the percentage of those living in proximity to conflict in Africa in the year 2000 can be considered low, at between 4% and 10% in all regions, the highest being in the North. However, by 2012 this range is between 9% and 30% with the Northern and Southern regions particularly badly affected. This situation has deteriorated further by 2014, in all regions apart from the South, with a range of between 10% and 29% of population in proximity to conflict across Africa.

### 4.4. Summary

Global, harmonised, geospatial datasets are important for consistent and standardised inputs for any type of modelling or comparison effort. This can include any number of discipline-specific foci including health, ecology, climate, and so on. The output of the work described here provides a valuable resource for both applied and research oriented efforts where challenges with data access, quality and consistency are low. These data products are important in providing consistency in application across countries, in order to achieve or monitor progress towards a variety of Sustainable Development Goals (SDGs). Further, these data products are important in providing consistency in the application within countries, which is arguably as important. Monitoring progress towards SDG achievement at sub-national scales (via assessment of health and socio-economic development metrics) relies on the acquisition of ongoing spatially detailed sub-national scale data on population counts and distributions (Tatem, 2017). It is therefore important to improve the availability of and access to disaggregated data and statistics. There is a need to take urgent steps to improve the quality, coverage and availability of disaggregated data in order to target interventions and ensure that no one is left behind (UN General Assembly, 2015). The applied examples, detailed in sections 3.2 and 3.3, demonstrate the potential usefulness of multi-temporal gridded population data at the subnational level, for use in the monitoring of health and development metrics.

## 5. Usage notes

Future global high-resolution population mapping can use these unique, open access, geospatial datasets to construct consistent and comparable, freely available, and potentially age-structured, annual high-resolution global population distribution layers for the 2000–2020 period, perhaps using methods for temporal considerations described by Gaughan et al. (2016). Future methods can involve fine-tuning of covariates used as input to an RF (or other type of) model, utilising a covariate selection optimised per region, continent, or globally as per user requirements. Some users may wish to produce population distribution datasets avoiding the use of certain geospatial datasets discussed in this paper, in order to avoid any endogeneity within their own research. The geospatial datasets can perhaps be improved upon in the future via use of updated OSM data, the replacement of existing OSM layers (which are time invariant) with newly harmonised multi-temporal datasets where appropriate, and/or replacement of datasets with those of higher spatial resolution, as they become available. Similarly, other multi-temporal datasets which correlate well with population density would be valuable additions to the archive, as would updates to existing annual layers. Suitable additions for population analysis might include agricultural layers (seasonal variation may be needed for migration predictions), the location of conflict zones (which disperse population) or the location of major employers/industries in rural areas (which gather working population). Further, potential exists in terms of measuring the impact of the downscaling of covariate datasets upon population and built settlement growth models. Five out of 11 source datasets: ESA CCI land cover (9”), DMSP nightlights (30”), ViiRS nightlights (15”), WDPA (30”), and WorldClim 2.0 (30”) have been downsampled to make the spatial resolution common. In an RF-based dasymetric model, the impact of this downsampling on predicted values could be lower as the model is trained on the mean of the aggregated data. However, for a built settlement model, because the model is trained on disaggregated pixel values of the selected samples and covariates, the impact could be higher. If downsampled covariates have a higher importance in model training then the impact of downsampling on predicted values may also be analysed in terms of sample size and sampling strategy.

Limitations of the geospatial dataset gridding process include the potential for small islands to be absent from the country ID base grid because the islands are not present in source CIESIN data. This has the consequence that corresponding small island topographic or other spatial data are excluded from the harmonised geospatial layers. Further, where coastlines differ between L0 country ID and input topography/other spatial layers, coastal pixels (with a data value) may be removed from the output grid during harmonisation. When linking the census table to the L1 census unit raster, it has been found that a few administrative units are smaller than the resolution of the raster. In such instances, in order to preserve population counts, the administrative unit is removed from the table and the corresponding population count added to that belonging to the neighbouring (larger) administrative unit as defined by the respective pixel in the raster. Further to this, population estimate interpolation/forecast do not take into account natural disasters or similar events. This is justified by the global and temporal extent of the study.

## Data Availability

The harmonised geospatial layers discussed in this paper are the product of the “Global High-Resolution Population Denominators Project” (WorldPop (www.worldpop.org) School of Geography and Environmental Science – University of Southampton, Department of Geography and Geosciences – University of Louisville, Département de Géographie – Université de Namur, & Center for International Earth Science Information Network (CIESIN) – Columbia University, 2018). Layers are being made publically available via the WorldPop FTP server (ftp://ftp.worldpop.org.uk/GIS/Covariates/Global_2000_2020/0_Mosaicked/), where licensing permits, reposited at the University of Southampton (https://doi.org/10.5258/SOTON/WP00650). Data is made available in the GeoTIFF format, with global coverage where source data permits.

## Supporting information

The following supporting information is available as part of the online article: Supplementary material S1. Methods elucidation: OSM highways, highway intersections, ESA CCI land cover; Results: Additional tables.

## Supplementary material

Supplemental data for this article can be assessed here.
