# Supplementary material for: Global spatio-temporally harmonised datasets for producing high-resolution gridded population distribution datasets
Source: Big Earth Data. 2019 Jun 18;3(2):108–39. doi: 10.1080/20964471.2019.1625151 (PMC6743742; doi:10.1080/20964471.2019.1625151)
Supplement: Supplemental Material [file TBED_A_1625151_SM5772.zip › supplementary material.docx]

**Supplementary Material**

**Methodology**

### **OpenStreetMap (OSM)**

**OSM highways**

| **Priority Value** | **Highway Tag** |
| --- | --- |
| 1 | 'proposed' OR 'construction' |
| 2 | 'path' OR 'footway' |
| 3 | 'track' OR 'bridleway' OR 'disused' OR 'unsurfaced' OR 'abandoned' OR 'trail' OR 'byway' OR 'unknown' OR 'unmarked_route' |
| 4 | 'service' OR 'services' |
| 5 | 'living_street' OR 'pedestrian' |
| 6 | 'unclassified' OR 'road' OR 'yes' |
| 7 | 'residential' |
| 8 | 'tertiary_link' |
| 9 | 'tertiary' |
| 10 | 'secondary_link' |
| 11 | 'secondary' |
| 12 | 'primary_link' |
| 13 | 'primary' |
| 14 | 'trunk_link' |
| 15 | 'trunk' |
| 16 | 'motorway_link' |
| 17 | 'motorway' |
| 30 | *Inter-coast bridges and tunnels (links) identified separately using ‘bridge’ and ‘tunnel’ tags* |

Supplementary Table 1. Priority values for OSM highway tags in the spatialite table.

OSM highway tags are assigned a priority value in the spatialite spatial relational table in order to simplify the complex tagging of highway data in the OSM database. A higher value represents a higher priority. Assignment of priority values makes highway tagging more manageable for display in raster format. Variants and/or misspellings of highway tags are included in assignment of priority values for completeness where applicable, but are not included in the table for reasons of brevity.

**OSM highway intersections**

The limitations of our intersection identification approach are that a limited number of genuine road intersections will be removed where bridge/tunnel road sections intersect (defined by a change in road name, reference number, junction, or priority tag) directly with a different highway. Also, where massively intertwined (multi-level, bridge) road traffic interchanges or complex underground highway tunnel networks occur then valid intersections will be omitted entirely. These are relatively trivial and/or spatially isolated errors of omission relative to the many errors of commission that would occur in the intersection layer were the method not to be applied, especially once the spatial resolution of the rasterized grid is considered. A future iteration of our methodology will involve deriving intersection by level, layer and/or location tags in order to remove the described errors.

To further elucidate the production methodology (using PostGIS functions in the GDAL ogr2ogr utility) of the intersection layer - highways with a priority greater than or equal to 8 in the spatialite table are identified and separately dissolved (i.e. point set union) by road name, reference number, junction, and priority using the ST_Unaryunion and ST_Collect functions. The ST_Intersection function is used to return the shared portion of each dissolved geometry. Intersection points are extracted from these layers and merged to form a provisional intersection layer.

Returning to the highway spatialite table, sections of highway are identified that have a priority greater than or equal to 8 and that are tagged as bridges or tunnels. Similar sections of highway that are specifically not bridges or tunnels are also identified. The ST_Intersection function is used to identify shared portions of lines between the two groups. Extracted points express only those intersections belonging to sections of highway defined as bridges and tunnels (i.e. where there are false intersections). The ST_Intersects function is subsequently used to remove such false intersections from the provisional intersection layer, producing the final highway intersection layer. Technical functions described in this section are described in detail in PostGIS PSC (2017a, 2017b).

**ESA CCI land cover**

| **Class** | **Landcover type** |
| --- | --- |
| 11 | Cropland Natural Vegetation |
| 40 | Tree Cover |
| 130 | Shrubland |
| 140 | Herbaceous Cover, Grassland, Mosses |
| 150 | Sparse Vegetation |
| 160 | Tree/ Herbaceous Cover, Flooded, Fresh/Saline/Brackish Water |
| 190 | Urban Areas |
| 200 | Bare Areas |
| 210 | Water Bodies, Permanent Snow and Ice |

Supplementary Table 2. ESA CCI aggregated reclassifications of land cover types.

In order to create an annual global land cover time series for 2000-2015, land use sub-category classifications are extracted and simplified (to 9 classes) for each annual input grid.

| **Results** |  | |  | |  | |  | |  | |  |  | | |  | |  | | |  |
| --- | --- | --- | --- | --- | --- | --- | --- | --- | --- | --- | --- | --- | --- | --- | --- | --- | --- | --- | --- | --- |
| **Country code** | | **Country code** | | **Country** | | | | **Total population (count) 2000** | | **Total population (count) 2014** | | | **Total population (count) affected where PfPR >10%, 2000** | | | **Total population (count) affected where PfPR >10%, 2014** | **Percentage of total population affected where PfPR >10%, 2000** | **Percentage of total population affected where PfPR >10%, 2014** |  |  |
| 12 | | DZA | | Algeria, People's Democratic Republic of | | | | 26,538,882 | | 34,024,856 | | | NULL | | | NULL | NULL | NULL |  |  |
| 24 | | AGO | | Angola, Republic of | | | | 12,352,447 | | 15,643,980 | | | 12,101,844 | | | 7,494,267 | 97.971 | 47.905 |  |  |
| 72 | | BWA | | Botswana, Republic of | | | | 659,731 | | 986,072 | | | 485 | | | 0 | 0.074 | 0.000 |  |  |
| 108 | | BDI | | Burundi, Republic of | | | | 6,652,640 | | 9,510,595 | | | 5,983,468 | | | 6,501,587 | 89.941 | 68.362 |  |  |
| 120 | | CMR | | Cameroon, Republic of | | | | 11,269,655 | | 19,209,423 | | | 11,099,439 | | | 17,959,665 | 98.490 | 93.494 |  |  |
| 132 | | CPV | | Cape Verde, Republic of | | | | 421,167 | | 474,071 | | | NULL | | | NULL | NULL | NULL |  |  |
| 140 | | CAF | | Central African Republic | | | | 1,732,214 | | 2,690,580 | | | 1,732,214 | | | 2,676,563 | 100.000 | 99.479 |  |  |
| 148 | | TCD | | Chad, Republic of | | | | 2,446,368 | | 5,855,398 | | | 1,849,065 | | | 5,295,088 | 75.584 | 90.431 |  |  |
| 174 | | COM | | Comoros, Union of the | | | | 529,832 | | 769,374 | | | NULL | | | NULL | NULL | NULL |  |  |
| 175 | | MYT | | Mayotte | | | | 151,696 | | 225,590 | | | NULL | | | NULL | NULL | NULL |  |  |
| 178 | | COG | | Congo, Republic of the | | | | 2,870,118 | | 1,861,044 | | | 2,823,997 | | | 1,832,134 | 98.393 | 98.447 |  |  |
| 180 | | COD | | Congo, Democratic Republic of the | | | | 30,719,535 | | 66,634,930 | | | 28,966,044 | | | 60,703,047 | 94.292 | 91.098 |  |  |
| 204 | | BEN | | Benin, Republic of | | | | 4,724,412 | | 8,708,180 | | | 4,724,043 | | | 8,479,070 | 99.992 | 97.369 |  |  |
| 226 | | GNQ | | Equatorial Guinea, Republic of | | | | 509,190 | | 703,167 | | | 243,944 | | | 363,672 | 47.908 | 51.719 |  |  |
| 231 | | ETH | | Ethiopia, Federal Democratic Republic of | | | | 53,154,081 | | 81,102,666 | | | 6,856,936 | | | 6,313 | 12.900 | 0.008 |  |  |
| 232 | | ERI | | Eritrea, State of | | | | 1,921,041 | | 2,511,033 | | | 182,215 | | | NULL | 9.485 | NULL |  |  |
| 262 | | DJI | | Djibouti, Republic of | | | | 471,933 | | 703,352 | | | 15,697 | | | 0 | 3.326 | 0.000 |  |  |
| 266 | | GAB | | Gabon, Gabonese Republic | | | | 548,170 | | 1,195,953 | | | 524,874 | | | 1,153,387 | 95.750 | 96.441 |  |  |
| 270 | | GMB | | Gambia, Republic of the | | | | 1,138,145 | | 1,866,165 | | | 781,615 | | | 25,989 | 68.674 | 1.393 |  |  |
| 288 | | GHA | | Ghana, Republic of | | | | 16,254,665 | | 25,551,912 | | | 16,248,182 | | | 25,445,179 | 99.960 | 99.582 |  |  |
| 324 | | GIN | | Guinea, Republic of | | | | 4,744,996 | | 7,179,947 | | | 4,013,495 | | | 6,054,598 | 84.584 | 84.326 |  |  |
| 384 | | CIV | | Côte d'Ivoire, Republic of | | | | 11,332,115 | | 18,010,638 | | | 11,331,920 | | | 18,009,657 | 99.998 | 99.995 |  |  |
| 404 | | KEN | | Kenya, Republic of | | | | 26,391,181 | | 41,284,699 | | | 20,339,651 | | | 26,293,247 | 77.070 | 63.688 |  |  |
| 426 | | LSO | | Lesotho, Kingdom of | | | | 1,487,711 | | 1,414,667 | | | NULL | | | NULL | NULL | NULL |  |  |
| 430 | | LBR | | Liberia, Republic of | | | | 1,663,287 | | 2,745,213 | | | 1,377,131 | | | 2,266,612 | 82.796 | 82.566 |  |  |
| 434 | | LBY | | Libyan Arab Jamahiriya | | | | 3,184,998 | | 4,312,746 | | | NULL | | | NULL | NULL | NULL |  |  |
| 450 | | MDG | | Madagascar, Republic of | | | | 9,442,466 | | 15,945,834 | | | 4,812,187 | | | 3,634,080 | 50.963 | 22.790 |  |  |
| 454 | | MWI | | Malawi, Republic of | | | | 10,144,836 | | 15,194,901 | | | 10,099,325 | | | 14,454,571 | 99.551 | 95.128 |  |  |
| 466 | | MLI | | Mali, Republic of | | | | 4,644,363 | | 11,238,721 | | | 4,625,936 | | | 11,183,226 | 99.603 | 99.506 |  |  |
| 478 | | MRT | | Mauritania, Islamic Republic of | | | | 1,073,385 | | 2,075,962 | | | 351,759 | | | 93,816 | 32.771 | 4.519 |  |  |
| 504 | | MAR | | Morocco, Kingdom of | | | | 24,757,932 | | 29,794,291 | | | NULL | | | NULL | NULL | NULL |  |  |
| 508 | | MOZ | | Mozambique, Republic of | | | | 9,699,320 | | 17,888,505 | | | 9,280,373 | | | 17,334,147 | 95.681 | 96.901 |  |  |
| 516 | | NAM | | Namibia, Republic of | | | | 1,179,435 | | 1,521,456 | | | 665,597 | | | 455,778 | 56.434 | 29.957 |  |  |
| 562 | | NER | | Niger, Republic of | | | | 6,358,253 | | 14,037,984 | | | 6,351,128 | | | 14,009,442 | 99.888 | 99.797 |  |  |
| 566 | | NGA | | Nigeria, Federal Republic of | | | | 107,916,690 | | 172,278,605 | | | 101,172,001 | | | 166,212,358 | 93.750 | 96.479 |  |  |
| 624 | | GNB | | Guinea-Bissau, Republic of | | | | 613,376 | | 996,650 | | | 589,416 | | | 270,175 | 96.094 | 27.108 |  |  |
| 646 | | RWA | | Rwanda, Republic of | | | | 7,740,593 | | 11,157,602 | | | 5,928,319 | | | 654,526 | 76.587 | 5.866 |  |  |
| 678 | | STP | | São Tomé and Príncipe, Democratic Republic of | | | | 116,531 | | 178,540 | | | NULL | | | NULL | NULL | NULL |  |  |
| 686 | | SEN | | Senegal, Republic of | | | | 7,148,586 | | 11,925,744 | | | 4,929,414 | | | 595,751 | 68.956 | 4.996 |  |  |
| 694 | | SLE | | Sierra Leone, Republic of | | | | 3,413,471 | | 4,958,526 | | | 2,812,696 | | | 4,078,608 | 82.400 | 82.254 |  |  |
| 706 | | SOM | | Somalia, Somali Republic | | | | 1,980,319 | | 3,618,877 | | | 836,020 | | | 498,033 | 42.216 | 13.762 |  |  |
| 710 | | ZAF | | South Africa, Republic of | | | | 42,216,548 | | 52,446,755 | | | 3,263,601 | | | 1,173,945 | 7.731 | 2.238 |  |  |
| 716 | | ZWE | | Zimbabwe, Republic of | | | | 6,460,182 | | 8,003,816 | | | 136,688 | | | 107,758 | 2.116 | 1.346 |  |  |
| 728 | | SSD | | South Sudan | | | | 965,591 | | 3,748,197 | | | 965,591 | | | 3,672,690 | 100.000 | 97.986 |  |  |
| 729 | | ESH | | Western Sahara | | | | 12,640,416 | | 22,262,637 | | | 2,763,654 | | | 483,870 | 21.864 | 2.173 |  |  |
| 732 | | SDN | | Sudan, Republic of | | | | 356,423 | | 420,609 | | | NULL | | | NULL | NULL | NULL |  |  |
| 748 | | SWZ | | Swaziland, Kingdom of | | | | 670,773 | | 755,779 | | | 873 | | | 18 | 0.130 | 0.002 |  |  |
| 768 | | TGO | | Togo, Togolese Republic | | | | 3,800,028 | | 6,304,188 | | | 3,799,930 | | | 5,909,660 | 99.997 | 93.742 |  |  |
| 788 | | TUN | | Tunisia, Tunisian Republic | | | | 8,000,785 | | 9,258,397 | | | NULL | | | NULL | NULL | NULL |  |  |
| 800 | | UGA | | Uganda, Republic of | | | | 21,734,584 | | 33,533,793 | | | 20,937,230 | | | 29,802,664 | 96.331 | 88.874 |  |  |
| 818 | | EGY | | Egypt, Arab Republic of | | | | 64,822,702 | | 83,991,958 | | | NULL | | | NULL | NULL | NULL |  |  |
| 834 | | TZA | | Tanzania, United Republic of | | | | 25,241,411 | | 40,607,860 | | | 23,441,621 | | | 16,577,620 | 92.870 | 40.824 |  |  |
| 854 | | BFA | | Burkina Faso | | | | 7,284,960 | | 14,418,077 | | | 7,284,960 | | | 14,415,691 | 100.000 | 99.983 |  |  |
| 894 | | ZMB | | Zambia, Republic of | | | | 4,237,089 | | 8,130,420 | | | 4,235,354 | | | 7,298,965 | 99.959 | 89.774 |  |  |
|  |  | |  | |  | |  | |  | |  |  | | |  | |  | | |  |
|  |  | |  | |  | |  | |  | |  |  | | |  | |  | | |  |
|  | | | | | |  | | | | | | | |  | | | | | | |
|  |  | |  | |  | |  | |  | |  |  | | |  | |  | | |  |
|  |  | |  | |  | |  | |  | |  |  | | |  | |  | | |  |
|  |  | |  | |  | |  | |  | |  |  | | |  | |  | | |  |
|  |  | |  | |  | |  | |  | |  |  | | |  | |  | | |  |
|  |  | |  | |  | |  | |  | |  |  | | |  | |  | | |  |
|  |  | |  | |  | |  | |  | |  |  | | |  | |  | | |  |
|  |  | |  | |  | |  | |  | |  |  | | |  | |  | | |  |
|  |  | |  | |  | |  | |  | |  |  | | |  | |  | | |  |
|  |  | |  | |  | |  | |  | |  |  | | |  | |  | | |  |
|  |  | |  | |  | |  | |  | |  |  | | |  | |  | | |  |
|  | | | | | |  | | | | | | | |  | | | | | | |

Supplementary Table 3. Country population affected by Plasmodium falciparum malaria infection in 2000 and 2014 where prevalence is >10%

| **Africa UN Zone** | **Region** | **Total population (count) 2000** | **Total population (count) 2012** | **Total population (count) 2014** | **Total population (count) within conflict zones, 2000** | **Total population (count) within conflict zones, 2012** | **Total population (count) within conflict zones, 2014** | **Percentage of regional population within conflict zones, 2000** | **Percentage of regional population within conflict zones, 2012** | **Percentage of regional population within conflict zones, 2014** |
| --- | --- | --- | --- | --- | --- | --- | --- | --- | --- | --- |
| 1 | North | 140,302,138 | 177,215,498 | 184,065,494 | 14,000,888 | 34,000,134 | 47,288,896 | 9.979 | 19.186 | 25.691 |
| 2 | East | 186,918,795 | 274,566,842 | 293,937,114 | 11,700,094 | 23,805,444 | 28,423,579 | 6.259 | 8.670 | 9.670 |
| 3 | Central | 62,564,228 | 102,907,908 | 113,973,015 | 3,913,139 | 13,170,507 | 18,368,842 | 6.255 | 12.798 | 16.117 |
| 4 | West | 182,531,899 | 281,903,921 | 302,770,583 | 9,489,334 | 30,894,837 | 42,992,579 | 5.199 | 10.959 | 14.200 |
| 5 | South | 46,214,198 | 55,325,676 | 57,124,729 | 1,876,689 | 16,499,427 | 16,519,103 | 4.061 | 29.822 | 28.918 |

Supplementary Table 4. African regional population affected by conflict in 2000, 2012, and 2014

**Supplementary References**

PostGIS PSC (Project Steering Committee). "PostGIS 2.4.4dev Manual - Chapter 8. PostGIS Reference - 8.9. Spatial Relationships and Measurements." Accessed March 2018. https://postgis.net/docs/reference.html#Spatial_Relationships_Measurements.

———. "PostGIS 2.4.4dev Manual - Chapter 8. PostGIS Reference - 8.11. Geometry Processing." Accessed March 2018. https://postgis.net/docs/reference.html#Geometry_Processing.
